# Lateral inhibition: Two modes of non-autonomous negative autoregulation by *neuralized*

**DOI:** 10.1371/journal.pgen.1007528

**Published:** 2018-07-20

**Authors:** Steven W. Miller, James W. Posakony

**Affiliations:** Division of Biological Sciences, Section of Cell & Developmental Biology, University of California San Diego, La Jolla, California, United States of America; Stanford University School of Medicine, UNITED STATES

## Abstract

Developmental patterning involves the progressive subdivision of tissue into different cell types by invoking different genetic programs. In particular, cell-cell signaling is a universally deployed means of specifying distinct cell fates in adjacent cells. For this mechanism to be effective, it is essential that an asymmetry be established in the signaling and responding capacities of the participating cells. Here we focus on the regulatory mechanisms underlying the role of the *neuralized* gene and its protein product in establishing and maintaining asymmetry of signaling through the Notch pathway. The context is the classical process of “lateral inhibition” within *Drosophila* proneural clusters, which is responsible for distinguishing the sensory organ precursor (SOP) and non-SOP fates among adjacent cells. We find that *neur* is directly regulated in proneural clusters by both proneural transcriptional activators and *Enhancer of split* basic helix-loop-helix repressors (bHLH-Rs), via two separate cis-regulatory modules within the *neur* locus. We show that this bHLH-R regulation is required to prevent the early, pre-SOP expression of *neur* from being maintained in a subset of non-SOPs following SOP specification. Lastly, we demonstrate that Neur activity in the SOP is required to inhibit, in a cell non-autonomous manner, both *neur* expression and Neur function in non-SOPs, thus helping to secure the robust establishment of distinct cell identities within the developing proneural cluster.

## Author summary

Much of the process of animal development is concerned with giving cells specific instructions as to what type of cell they are to become—their “fate”. Often, it is even necessary to assign very different fates to cells that are adjacent to each other in the tissue. In such cases, cell-to-cell signaling is frequently utilized as the means of distinguishing the cells’ fates. For example, one cell might send a signal to its neighbors that inhibits them from adopting the same fate as itself. Here, it is obviously vital that there is an asymmetry between the “sending” and “receiving” cells in the ability to transmit such a signal. In the fruit fly *Drosophila*, the gene *neuralized* encodes a protein that plays a critical role in establishing the capacity to send such an inhibitory signal. The work we describe here reveals specifically how the receiving cells are prevented from acquiring the ability to send the signal. Remarkably, the Neuralized protein itself is deeply involved in this process. Neuralized function in the sending cell generates two distinct mechanisms that inhibit its own activity in the receiving cells.

## Introduction

The specification of discrete cell identities during metazoan development often requires the establishment of disparate genetic programs in adjacent cells. The Notch signaling pathway is ideally suited to this task, since it mediates direct cell-cell interactions via contact between transmembrane ligands and receptors. Acting in this fashion, it is responsible for distinguishing the gene expression programs of adjacent cells in multiple developmental settings, including boundary formation between neighboring cell populations; binary cell fate specification between daughter cells in a cell lineage; and “lateral inhibition” within a cluster of cells with initially similar fate [1].

If such binary partitioning of cell fate is to function with high fidelity, it ultimately requires the creation of strong disparities in Notch signaling and responding capacity between “sending” and “receiving” cells. In principle, this can be achieved in a number of ways, most obviously via differences in ligand and/or receptor protein levels [2]. In contexts in which such differences are not observed, however, other mechanisms must come into play. One example is the classical process of lateral inhibition within proneural clusters (PNCs) in *Drosophila*.

The cells that comprise the mechanosensory bristles of *Drosophila* are products of serial asymmetric cell divisions, beginning with individual sensory organ precursor cells (SOPs) that are specified by Notch signaling within PNCs. PNCs are defined by the expression of basic helix-loop-helix (bHLH) transcriptional activators, encoded by the “proneural” genes *achaete* (*ac*) and *scute* (*sc*), that confer upon PNC cells the potential to adopt the SOP fate [3, 4]. Due in part to their positive auto-regulatory activity, the expression of proneural genes is elevated in cells that will become SOPs.

SOPs use Notch signaling to inhibit neighboring PNC cells from becoming SOPs [5, 6]. Notch receptor on the surface of these “non-SOP” cells is activated by cell-surface ligand on the SOP, resulting in the release of the intracellular domain (ICD) of the receptor from the plasma membrane and its translocation to the nucleus. There, the Notch ICD forms a complex with the pathway’s transducing transcription factor Suppressor of Hairless [Su(H)], converting it from a repressor to an activator and stimulating the expression of a collection of SOP-inhibitory target genes [1].

The *Enhancer of split* [*E(spl)*] and *Bearded* (*Brd*) gene complexes encode two major classes of Notch effectors, the E(spl) bHLH transcriptional repressors (bHLH-Rs) and the Brd family members (BFMs) [7–11]. The bHLH-Rs prevent non-SOPs from becoming SOPs in part by reducing proneural auto-activation [12], and also by repressing transcription of SOP-specific genes [13]. BFMs function very differently—they bind directly to the E3 ubiquitin ligase Neuralized (Neur), thereby blocking its direct interaction with the ICDs of the Notch ligands Delta (Dl) and Serrate (Ser) [14, 15]. Neur expression is strongly upregulated in SOPs, and mono-ubiquitination of ligand ICDs by Neur promotes ligand endocytosis and their ability to activate the Notch receptor [16–18].

Central to the establishment and maintenance of the two distinct PNC cell fates is the emergence of an imbalance in Notch signaling capacity between the SOP and non-SOPs, despite the fact that all PNC cells express both ligand and receptor. *Dl* has been proposed as a direct target of the proneural proteins in neural precursor (NP) cells [19], which in principle could lead to upregulation of its expression specifically in SOPs. However, NP specification can proceed normally when *Dl* is uncoupled from proneural regulation [20, 21], and similar levels of nascent *Dl* transcript have been observed in microchaete SOPs and surrounding non-SOPs [22]. By contrast, direct proneural regulation of *neur* is an attractive alternative, because of the gene’s high SOP-specific expression and its important role in Notch-mediated lateral inhibition.

Prior investigations of Neur function in SOP specification have addressed neither the transcriptional regulation of *neur* nor the specific processes by which functional Neur activity is prevented in non-SOPs. Here we directly address the mechanisms by which Neur contributes to the establishment of unequal signaling capacity between SOPs and non-SOPs. In a previous report [13], we described the identification and functional activities of neur4D and neur1B, two enhancer modules that drive *neur* expression in NP cells. In the present study, we investigate the transcription factor inputs and regulatory logic that these modules use to generate the NP specificity. We demonstrate that *neur* is a direct target of both the proneural proteins and the bHLH-Rs, acting through the neur4D and neur1B enhancers. In particular, we identify a conserved proneural motif type that is capable of binding both the Ac/Sc and Atonal classes of proneural activators, and show that mutation of bHLH-R binding motifs causes expansion of both *neur* transcript and protein into non-SOP territories. We also provide conclusive evidence of nascent *neur* transcription in a small subset of PNC cells prior to SOP commitment. This analysis offers for the first time an explicit definition of the “*neur* group” of PNC cells [23], and resolves the previous apparent inconsistency of complementary expression patterns for *neur* and BFMs. Lastly, we demonstrate the consequences of either maintaining *neur* expression in non-SOPs or blocking Neur activity specifically in the SOP. Together, our work shows that, through its function in promoting Notch signaling from the SOP, *neur* auto-inhibits, in a cell non-autonomous manner, both its proneural-dependent transcription and the function of its product Neur, and by these mechanisms helps to establish and maintain an SOP/non-SOP dichotomy in signaling capacity.

## Results

### P_S_ motifs in the neur4D and neur1B modules are strongly conserved, but are not required for their activity

In the wing imaginal disc, both the accumulation of endogenous *neur* transcript and the expression of the *neur4D-GFP* and *neur1B-GFP* reporter transgenes are dependent upon proneural *ac/sc* gene activity in trans [13, 24]. Consistent with direct proneural regulation of neur4D, five Ac/Sc binding motifs fitting the RCAGSTG definition (which we refer to here as P_S_) are found in this module in *D*. *melanogaster*. Moreover, four of these five are fully conserved in 11 other *Drosophila* species, the exception being the P5 site in *D*. *mojavensis* and *D*. *virilis*, which is changed to RCAGATG, referred to here as P_A_ (S1A and S2A Figs). By contrast, in *D*. *melanogaster* neur1B, we find only a single P_S_ motif. This is conserved in 10/12 species, the exceptions being *D*. *persimilis* and *D*. *pseudoobscura*, in which the motif deviates to the P_A_ form (S1B and S2B Figs).

**Fig 1:**
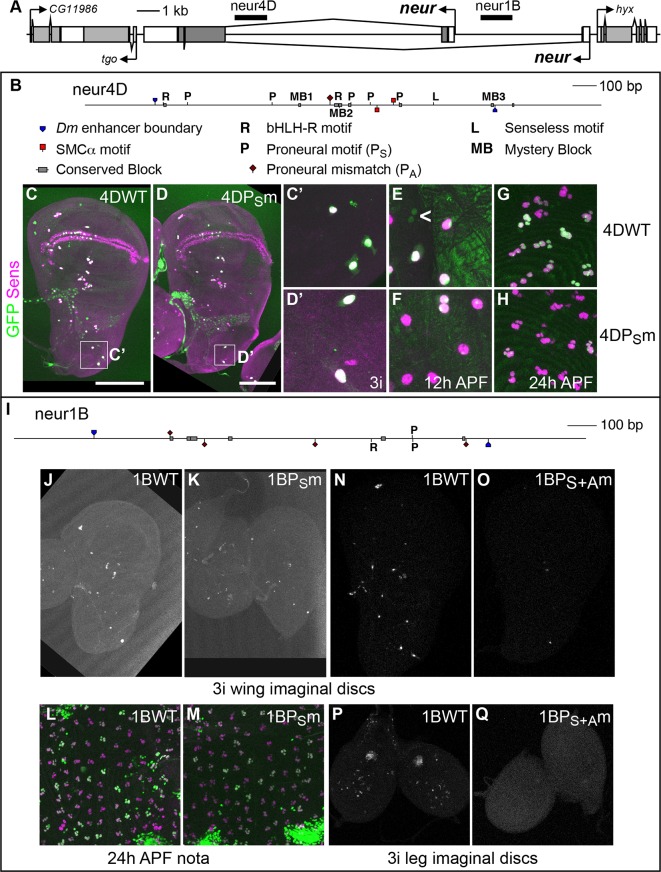
Two *neuralized* SOP enhancers contain conserved binding sites for both proneural proteins and bHLH repressors. (A) Diagram of the *neur* locus and flanking genes, showing the locations of the neur4D and neur1B SOP enhancers [13]. (B) Expanded diagram of the neur4D enhancer, marking the positions of proneural and bHLH-R binding motifs, along with other conserved sequences. (C-H) GFP expression (green) driven by a wild-type (WT) neur4D reporter construct (C, C’, E, and G) or by a proneural motif mutant (P_S_m) version (D, D’, F, and H) in representative third-instar wing imaginal discs (C-D’), 12 h APF nota (E and F), and 24 h APF nota (G and H); C’ and D’ show the scutellar and dorsocentral regions of the wing disc (see boxes in C and D). SOPs are marked by Sens protein (magenta). Caret (<) in (E) identifies two small, adjacent GFP-positive, Sens-negative nuclei. (I) Expanded diagram of the neur1B enhancer, showing the positions of proneural and bHLH-R binding motifs, along with other conserved sequence blocks; refer to (B) for symbol definitions. (J-Q) GFP expression driven a wild-type (WT) neur1B reporter construct (J, L, N, and P), a construct in which the the single P_S_-type proneural motif is mutated (P_S_m; K and M), and a construct in which both the P_S_- and P_A_-type proneural motifs are mutated (P_S+A_m; O and Q) in third-instar wing imaginal discs (J, K, N, and O), 24 h APF nota (L and M), and third-instar leg imaginal discs (P and Q). In panels L and M, GFP is in green, Sens protein in magenta. See also S1 and S2 Figs.

Given the overall strong conservation of the P_S_ motifs in neur4D and neur1B, we sought to assess their functional role *in vivo*. All P_S_ sites in each enhancer were changed from RCAGSTG to RAAGSGG, a mutation known to abrogate binding of Ac/Da heterodimers [25]. We observed only a slight reduction in GFP expression driven by both neur4D and neur1B (Fig 1C–1H and 1J–1M; S4B1, S4B8, S4B15 and S4B22 Fig). This result suggests two possibilities that are not mutually exclusive: First, that direct activation of the neur4D and neur1B modules by proneural factors is mediated by binding sites other than P_S_ motifs; second, that whatever the role of proneural proteins in direct activation of the two modules, other factors are sufficient to drive their activity in SOPs.

### Activation of the neur1B enhancer depends on P_A_, a variant proneural protein binding motif

Though *D*. *melanogaster* neur1B includes only a single match to the P_S_ motif definition, outside the *D*. *melanogaster-simulans-sechellia* sub-subgroup a second P_S_ motif occurs within this enhancer (S1B and S2B Figs). Interestingly, within the sub-subgroup this motif is changed to the P_A_ variant. A search of *D*. *melanogaster* neur1B and orthologous regions in the other species revealed the presence of three additional conserved CAGATG sequences (S1B and S2B Figs). The conservation of multiple P_A_ motifs and the switching of orthologous motifs from P_S_ to P_A_ within both neur4D and neur1B prompted us to ask whether the proneural proteins are capable of binding P_A_ motifs in an electrophoretic mobility shift assay (EMSA). Indeed, we find that Sc/Da heterodimers bind probes containing both the P_S_ and P_A_ motifs in neur1B, but not their corresponding mutant probes (S6A Fig). We next examined the consequences of mutating this expanded group of Ac/Sc-binding motifs, both P_S_ and P_A_, in the context of the *neur4D-GFP* and *neur1B-GFP* reporter transgenes.

There is a single P_A_ motif in *D*. *melanogaster* neur4D that is not present in *D*. *ananassae*, *D*. *mojavensis*, *D*. *virilis*, and *D*. *grimshawi* (S1A and S2A Figs), which may suggest that it is not required. Mutating this sequence in combination with the P_S_ motifs did not result in a further decrease in reporter expression (S6J, S6Q and S6R Fig). In contrast, and consistent with our EMSA data, mutation of both the P_S_ and P_A_ motifs in *neur1B-GFP* strongly reduced expression in the Ac/Sc-dependent proneural clusters of the wing imaginal disc (Fig 1O), suggesting that Ac/Sc proteins directly activate neur1B through these motifs. Interestingly, this reporter mutant also lost expression in both the ventral radius of the wing imaginal disc and the chordotonal clusters of the leg imaginal discs (Fig 1O and 1Q), territories in which the distantly related proneural protein Atonal (Ato) is active [26]. Consistent with this loss of expression, Ato has been reported to bind CAGATG sequences [26, 27], suggesting that Ato may also regulate neur1B through these P_A_ motifs. Indeed, we find that Ato/Da heterodimers are capable of binding all P_S_ and P_A_ motifs in neur1B *in vitro*, but not their mutant versions (S6A Fig).

### Activation of the neur4D enhancer is complex

Our results indicate a stark contrast in the requirements for proneural motifs in the activation of the neur4D and neur1B enhancers. Since the proneural motifs in neur4D are not strictly required for its activity, we sought to examine the conservation and functional necessity of other sequence elements within this module, some of which have previously been implicated in SOP-specific expression. In addition to the P_S_ and P_A_ motifs, the neur4D enhancer contains several motifs of at least seven nucleotides that are identical both in sequence and in order in all 12 *Drosophila* genomes (Fig 1B; S1A and S2A Figs). neur4D contains conserved instances of the SMCα motif [28–30]; the binding motif for the zinc-finger transcription factor Senseless (Sens) [31, 32]; and three other sequences that are fully conserved in all twelve genomes, which we refer to as “mystery blocks” (MB1, MB2, and MB3).

By mutational analysis, we examined the functional requirements for these conserved motifs in neur4D, both on their own and in combination with mutation of all the P_S_ motifs. Mutation of the two SMCα motifs alone only slightly reduced the activity of neur4D (S4A3, S4A10, S4A17 and S4A24 Fig). Mutating the SMCα motifs plus the P_S_ motifs further reduced reporter gene expression, but failed to eliminate it (S4B3, S4B10, S4B17 and S4B24 Fig). Similarly, modest reductions in neur4D activity were observed with mutation of either the Sens motif or MB2, whereas mutation of MB1 and MB3 each resulted in slightly increased expression (S4A2, S4A5–S4A7, S4A9, S4A12–S4A14, S4A16, S4A19–S4A21, S4A23 and S4A26–S4A28 Fig). We also assayed this series of mutant reporter genes by *in situ* hybridization with a *GFP* probe in embryos of various stages (S5 Fig). Similar to the results in larval and pupal tissues, no single motif mutation eliminated reporter expression. However, whenever the P_S_ motifs were also mutated (S4B Fig) we observed a consistent qualitative reduction in expression in comparison to the mutation of the motif classes individually. Since none of the motif mutants, whether on their own or in combination with the P_S_ mutations, eliminated neur4D activity, we made a construct in which all the sites contributing weak positive input (SMCα, Sens, MB2, and P_S_) were mutated. We observed weak GFP expression in wing imaginal discs even for this construct (S6K Fig), suggesting that still other sequences in neur4D play a role in its activation. Furthermore, even the addition of the P_A_ motif mutation to the P_S_+SMCα+Sens+MB2 mutant failed to yield any further reduction in wing disc expression driven by neur4D (S6L Fig). Thus, the SOP-specific activation of neur4D, in contrast to that of neur1B, appears to be highly complex and require inputs from other, as-yet-unknown factors.

### The conserved bHLH-R motifs in neur4D and neur1B prevent proneural-dependent activity in non-SOPs

While neur4D and neur1B exhibit a striking difference in their schemes for activation in SOPs, both enhancers contain one or more conserved motifs for binding by E(spl) bHLH repressor (bHLH-R) proteins (Fig 1; S1 and S2 Figs) [33]. These factors are expressed in a pattern complementary to that of *neur* in the PNC, due to default repression by Su(H) in the SOP and synergistic activation by the proneurals and Su(H) in non-SOPs (the “S+P” cis-regulatory code) [34, 35]. All three instances of the bHLH-R core binding motif (CACGYG) in neur4D and neur1B are conserved in all 12 genomes (S1 and S2 Figs).

**Fig 2:**
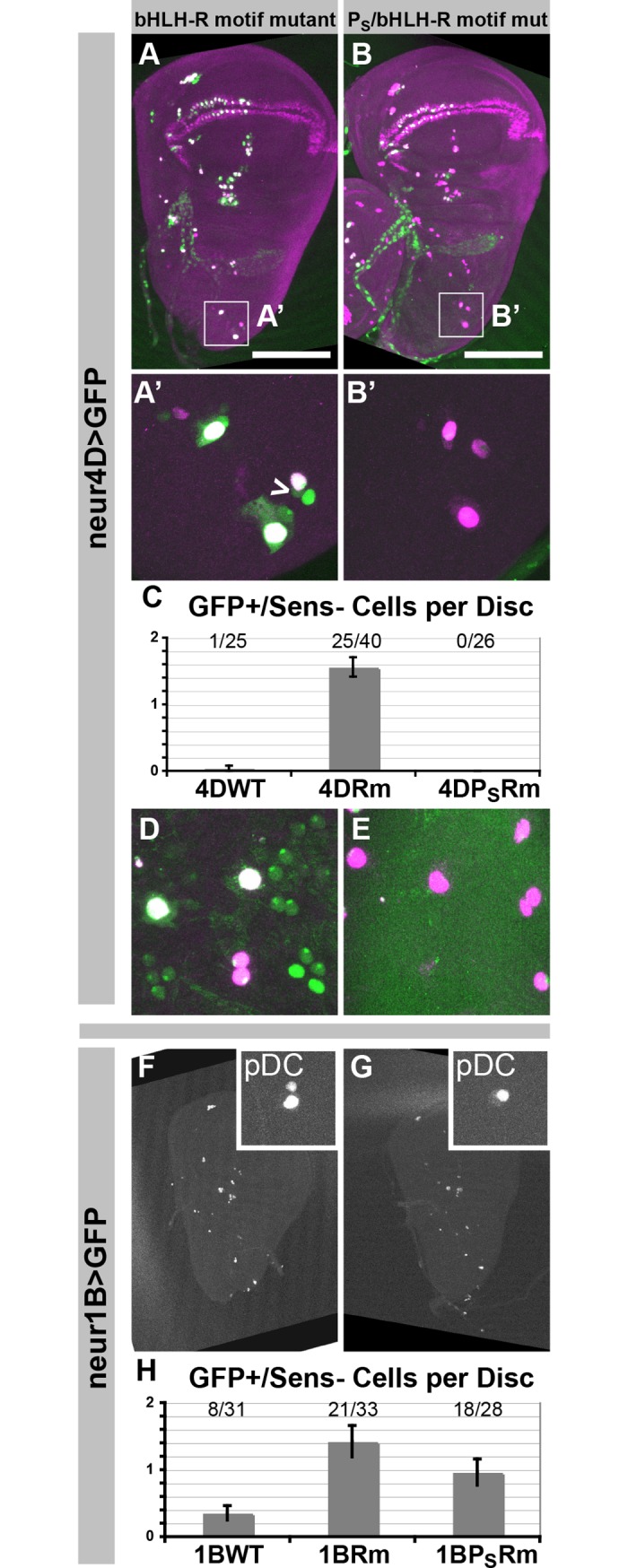
Mutation of bHLH repressor binding motifs in the neur4D and neur1B enhancers causes proneural motif-dependent ectopic reporter gene expression in non-SOPs. (A-B’, D, and E) Comparison of neur4DRm and neur4DP_S_Rm GFP reporter activities in third-instar wing imaginal discs (A-B’) and 12 h APF nota (D and E). (C) Quantification of ectopic GFP-expressing cells in the scutellar and dorsocentral macrochaete clusters in wing discs from larvae carrying the indicated reporter constructs. The proportion of discs exhibiting ectopic GFP is indicated for each genotype, and the graph reflects the average number of ectopic GFP cells over all discs. (F and G) Comparison of neur1BRm and neur1BP_S_Rm GFP reporter activities in wing discs. (H) Quantification of ectopic GFP-expressing cells adjacent to the posterior dorsocentral (pDC) macrochaete SOP cell in wing discs from larvae carrying the indicated reporter constructs. Graph presented as described for C. A’ and B’ show the scutellar and dorsocentral regions of the wing disc (see boxes in A and B); insets in F and G show only the region surrounding the pDC SOP. F and G show only the GFP signal; in the remaining images, GFP is in green and Sens protein is in magenta. Caret in A’ points to GFP-positive, Sens-negative cells; see text for details. Error bars in C and H represent standard error of the mean (SEM).

Based on both their pattern of expression and *cis-*regulatory logic, the bHLH-Rs would be predicted to confine *neur* expression to the SOP through the binding motifs in neur4D and neur1B. Indeed, when we mutate the two bHLH-R motifs in *neur4D-GFP*, we frequently observe many PNC positions in the wing imaginal disc where there is at least one GFP-positive cell in addition to the GFP- and Sens-positive SOP, usually located adjacent to the SOP (Fig 2A’ and 2C). Likewise, in the 12 hr APF notum we observe many regions in between Sens-positive SOPs that display multiple GFP-positive, Sens-negative cells (Fig 2D). We find that this ectopic GFP expression (outside of the SOP) is entirely dependent upon proneural cis-regulatory input via P_S_ sites in neur4D (Fig 2B’, 2C and 2E). This antagonistic functional relationship between bHLH-R and P_S_ motifs was also observed using *neur1B-GFP*. Mutation of the single bHLH-R motif in *neur1B-GFP* did not cause ectopic expression as broad as that seen by mutating the neur4D motifs; the position most regularly affected was the posterior dorsocentral. We frequently observed ectopic GFP expression at this position in *neur1BRm-GFP* wing discs, and it always appeared adjacent to the SOP (Fig 2F and 2H). Moreover, mutating the single P_S_ site in neur1B was sufficient to reduce this ectopic expression significantly (Fig 2G and 2H). These data demonstrate a functional requirement in both neur4D and neur1B for intact bHLH-R *cis*-regulatory input to confine the proneural-dependent activation of these enhancers to the SOP.

### Inhibiting bHLH-R binding to the *neur* SOP enhancers causes ectopic accumulation of *neur* transcript and protein

**Fig 3:**
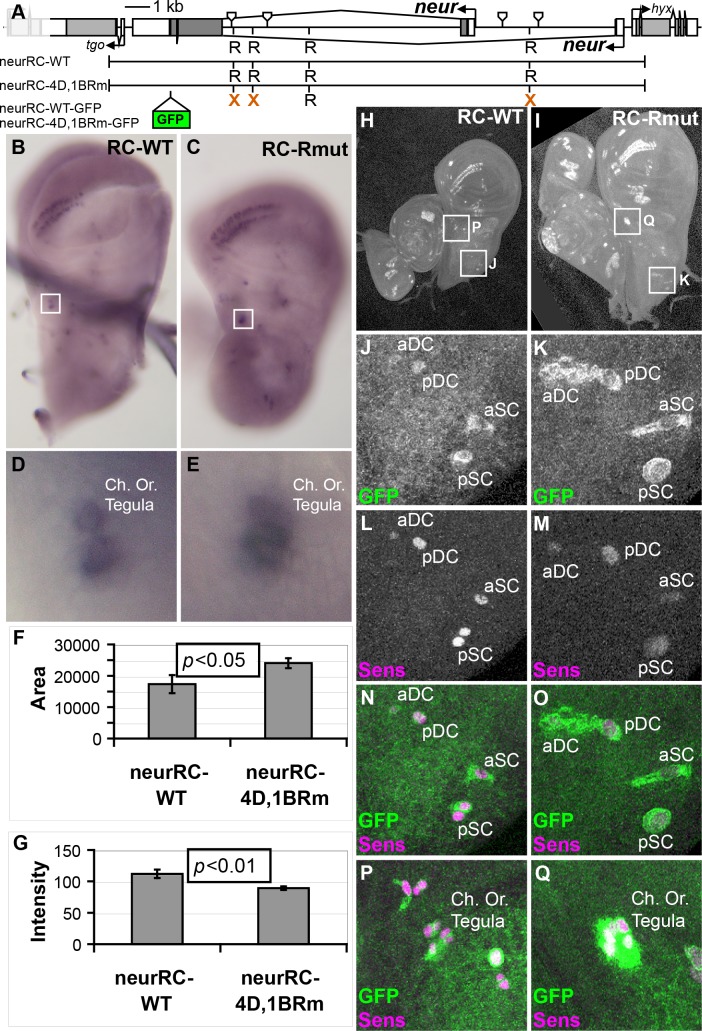
Mutation of bHLH repressor binding motifs in the 4D and 1B enhancer segments within a *neur* rescue construct causes ectopic expression of *neur*. (A) Diagram of the region surrounding the *neur* locus. Shown are the boundaries of neur4D and neur1B, the extent of the *neur* rescue constructs, the locations of bHLH-R binding motifs (those mutated in the rescue constructs are indicated by X’s), and the location of the GFP coding sequence in the tagged rescue constructs. (B-G) Comparison of *neur* transcript accumulation in wing imaginal discs from neurRC-WT (B and D) and neurRC-4D,1BRm (C and E) larvae. Boxes in B and C surround the developing chordotonal organ of the tegula, shown under higher magnification in D and E. (F) Quantification of the area of *neur* probe *in situ* hybridization signal over the chordotonal organ of the tegula [17340±2888 SEM (n = 9) vs. 24040±1575 SEM (n = 21)]. (G) Quantification of the white intensity over the same region, which is inversely proportional to the darkness of staining [112±6.68 SEM vs. 89.2±2.8 SEM]. (H-Q) Comparison of GFP signal in wing imaginal discs from neurRC-WT-GFP (H, J, L, N, and P) and neurRC-4D,1BRm-GFP (I, K, M, O, and Q) larvae. Boxes in H and I denote regions shown at higher magnification in the indicated panels. J and K show GFP signal alone; L and M shown Sens protein signal alone; N and O show the merged signals (GFP in green, Sens in magenta). P and Q are likewise merged images. aDC, pDC: anterior and posterior dorsocentral macrochaetes; aSC, pSC: anterior and posterior scutellar macrochaetes; Ch. Or.: chordotonal organ.

Because we had found, first, that both enhancers contribute to *neur* function in the SOP [13] and, second, that mutation of the bHLH-R input in both the *neur4DRm-GFP* and *neur1BRm-GFP* reporters causes ectopic expression in non-SOP cells, we sought to examine if this regulatory relationship can be observed in the context of the *neur* gene itself. To test this, we created both untagged and C-terminal GFP fusion versions of a wild-type P[acman] construct [36] containing 21 kb of the *neur* locus, extending into the adjacent genes, along with a variant in which the bHLH-R motifs within neur4D and neur1B are mutated (Fig 3A). Examining third-instar wing imaginal discs from larvae containing the untagged constructs, we saw an expansion of *neur* mRNA transcript expression, particularly at the wing margin and at the chordotonal organ of the tegula (Fig 3D and 3E). We quantified changes at this latter position using ImageJ software. Discs containing the bHLH-R motif mutant rescue constructs measured a statistically significant increase in the area of staining (Fig 3F), as well as a very significant decrease in average white intensity (Fig 3G), which is due to the increased darkness of the *in situ* signal. While these results clearly indicate an increase in *neur* transcript accumulation following disruption of bHLH-R-mediated repression, the spatial resolution of this assay is rather poor. A more conspicuous result was obtained using the GFP-tagged rescue constructs, with which we were regularly able to detect an expansion of Neur-GFP expression from the R motif mutant construct into more cells than just the specified SOPs (Fig 3K, 3O and 3Q). Together, these data demonstrate that mutation of the bHLH-R binding motifs within the two *neur* SOP enhancers results in the failure to confine *neur* transcript and protein to the SOP.

### “Pre-SOP” cells in the proneural cluster activate *neur*

The logic of confining a fully functional level of Neur protein accumulation to the SOP is clear: It is critical that only one cell in the proneural cluster should have the capacity to inhibit the SOP fate in all of its neighbors. However, the very reliance on proneural input (whether direct or indirect) to activate *neur* expression in SOPs creates the possibility that *neur* would initially be activated in more PNC cells than just the ultimate committed SOP. Consistent with this expectation, Huang *et al*. observed *neur* reporter gene (*neur*^*A101*^*-LacZ*) expression in 2–3 adjacent or nearby cells during macrochaete SOP specification [37]. Likewise, Koto *et al*. used a *neur-GAL4* driver to visualize the appearance of excess *neur*-positive cells during microchaete SOP determination [38]. We similarly have observed reporter gene (*neur4D-GFP*) expression in two adjacent cells prior to SOP specification, as determined by costaining with anti-Sens (Fig 1E; see caret).

**Fig 4:**
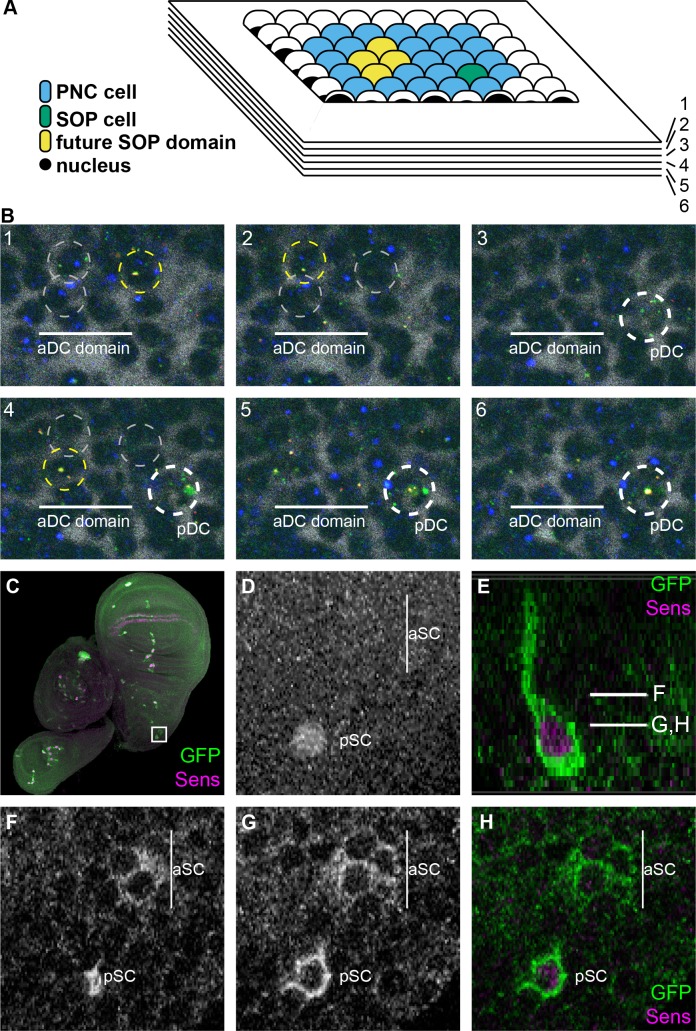
Expression of *neur* in PNC cells prior to SOP specification. (A) Diagram of heterochronic PNCs in the wing imaginal disc. Within such clusters (blue), one SOP (green) is specified before the other, which forms at a stereotypic position (yellow) a few cells away. (B) Multiplex fluorescent *in situ* hybridization with intron probes against *CG32150* (green only, marks a specified SOP), *sca* (blue, marks cells of the PNC), and *neur* (overlap of green and red). Hoescht stain, marking nuclei, is represented by inverted gray. Six adjacent 1-μm sections are shown from the dorsocentral (DC) macrochaete cluster of a wing imaginal disc. The *CG32150-*, *neur-*positive pDC SOP nucleus is marked with a white broken circle in panels 3–6. *neur*-positive nuclei in the nearby aDC domain are marked with yellow broken circles when the *neur* signal is present and gray broken circles when a different nucleus has *neur* signal. (C-H) Wing imaginal disc from a neurRC-WT-GFP larva showing GFP signal (C and E-H; green in C, E, and H) and Sens protein signal (C, D, E, and H; magenta in C, E, and H) in the heterochronic scutellar (SC) macrochaete cluster (region boxed in C, magnified in D-H). (D) Maximum projection of Sens signal. (E) Cross-section through the pSC nucleus, showing the locations of individual sections in the remaining panels. (F-H) GFP signal in individual confocal sections, showing at least four GFP-positive cells in the aSC domain, where Sens signal has yet to be strongly activated.

We sought more detailed documentation of this phenomenon by detection of either *neur* transcript or protein during the heterochronic appearance of macrochaete SOPs in the wing imaginal disc. In the notum region of the wing disc, these SOPs are first detected in a consistent temporal order [37]. Furthermore, certain of the individual clusters (e.g., dorsocentral and scutellar) develop exactly two SOPs, with one appearing early in development and the second appearing later in a stereotypical location a few nuclear diameters away (Fig 4A). This developmental pattern allowed us to fix larval imaginal discs at a stage in which a cluster contained both a specified early SOP and a nearby region, the “pre-SOP domain”. Indeed, in several of these heterochronic clusters we were able to find clear examples of *neur* expression in multiple adjacent cells by detecting either *neur* nascent transcript or GFP-tagged Neur protein (Fig 4B and 4F–4H).

For the former experiment, we utilized the multiplex fluorescent *in situ* hybridization technique [39] with intron probes to simultaneously visualize nascent transcripts for *neur*, *sca* (to mark PNC membership), and *CG32150* (to positively identify a committed SOP) [24], while also staining with Hoechst, a DNA dye to mark the nucleus. To be certain of the *neur* transcript detection, we used versions of the same *in situ* hybridization probe with two different labels simultaneously; thus, strong colocalization of these two probes unambiguously identifies cells producing *neur* nascent transcript. Regularly, within the dorsocentral (DC) and scutellar (SC) PNCs, one nucleus (the posterior cell) exhibited colocalization of strong *neur* probe signal in both channels, as well as a strong signal for *sca* and *CG32150* probes, identifying the first specified SOP in each of these clusters (Fig 4B, panels 5 and 6). In these same clusters, 1–3 nuclear diameters away, we were often able to find 2–4 cells that each colocalized *neur* probes (Fig 4B, panels 1, 2, and 4). In these cells, the probe density was not as strong as in the specified SOP, nor did these cells have strongly detectable *CG32150* transcript. When *CG32150* transcript was detected in this region, it was confined to a single nucleus that also exhibited *neur* probe colocalization at an increased density.

We also examined Neur protein accumulation in these pre-SOP domains, using a wild-type *neur* GFP-tagged rescue construct. Analogous to the *in situ* hybridization experiments, we co-stained with anti-Sens antibody to identify committed SOPs, and looked for Neur::GFP signal in a region a few cells away with no detectable Sens. Similar to what was seen in the *neur* transcript assay, we were able to detect 2–3 adjacent cells with GFP signal above background in these regions, typically in the DC and SC PNCs (Fig 4F–4H). Collectively, these data indicate that prior to demonstrated SOP commitment a subset of cells in the PNC express both *neur* transcript and protein.

### Maintaining *neur* expression in non-SOPs compromises lateral inhibition

**Fig 5:**
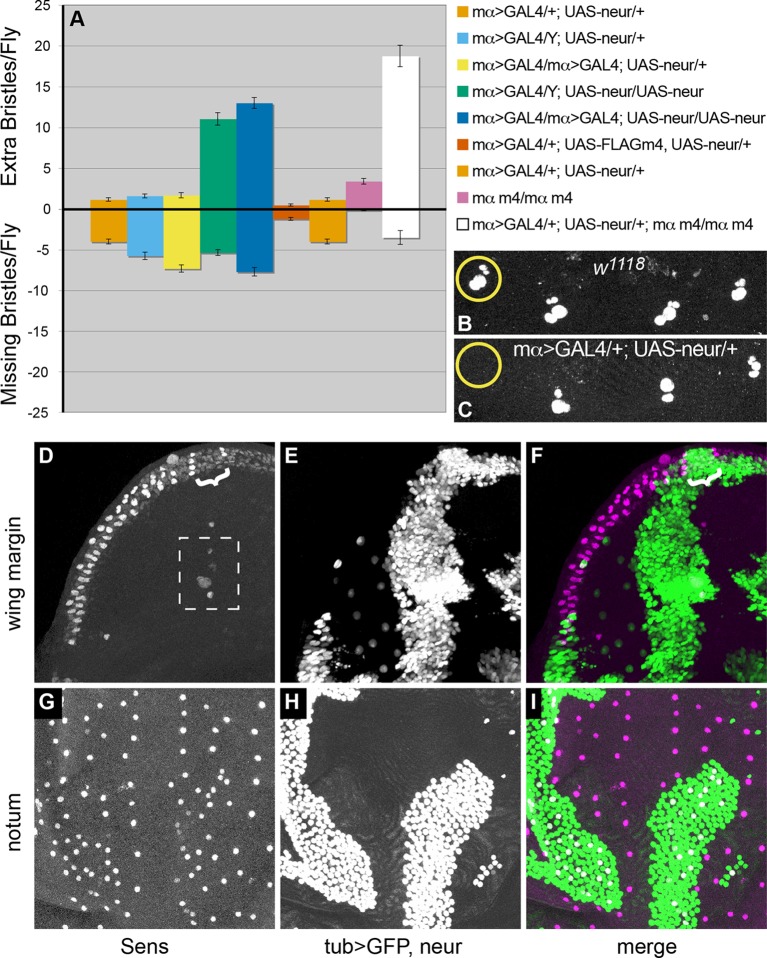
Forcing persistent non-SOP expression of *neur* causes both loss and gain of SOPs. (A) Quantification of macrochaete gain and loss on the dorsal head and thorax of flies of the genotypes indicated at right. Error bars represent SEM. (B and C) Scutellar bristle positions in 24 hr APF nota of the indicated genotypes, stained with anti-Cut antibody, show loss of the SOP with *neur* misexpression. (D-I) Uniform expression of *UAS-neur* driven by *tub-GAL4* in *neur* mutant clones using the MARCM system in either a wing imaginal disc (D-F) or a 12 hr APF notum (G-I). GFP (green in F and I) marks the territories of *tub>neur* expression; anti-Sens antibody signal (magenta in F and I) marks SOPs. Brackets in D and F mark SOP loss at the region of overlap between *tub>neur* activity and the wing margin. Sens-positive cells boxed in D are in a different focal plane from the GFP-expressing cells.

As we have seen, *neur* expression is ultimately tightly restricted to the SOP, yet prior to specification it occurs in more than one cell. We sought to investigate the potential consequences of persistent *neur* expression outside of the single committed SOP. Enhanced Notch signaling due to ectopic Neur expression in non-SOPs could conceivably interfere with proper lateral inhibition in two main ways. First, it could lead to loss of normal SOPs by preventing or overcoming their commitment to this fate. Alternatively, it could allow multiple cells in the PNC to resist signaling from the SOP and become committed SOPs themselves (perhaps due to cis-inhibition [40]). To explore these possibilities, we utilized two different strategies to misexpress *neur* and looked for manifestations of either of the predicted phenotypes. We first expressed Neur specifically in non-SOPs within the PNC using the non-SOP-specific, Notch-dependent driver *mα-GAL4*. In flies bearing single copies of both the driver and *UAS-neur*, the dominant phenotype was missing bristles (Fig 5A), which we confirmed to have resulted from loss of the SOP (Fig 5C). Adding an additional copy of the driver primarily enhanced SOP loss, while adding an additional copy of the responder significantly increased the number of extra bristles (Fig 5A). One complication of this strategy for misexpression is the fact that the *E(spl)mα* regulatory region is Notch-regulated [35]. Thus, if Notch signal receipt in non-SOPs is compromised, the expression of GAL4 could accordingly decrease. We therefore sought to examine the consequences of Notch-independent, uniform *neur* expression in mosaic tissue using the MARCM system [41]. Similar to the *mα-GAL4* experiments, we observed both SOP loss (Fig 5D–5F) and gain (Fig 5G–5I), depending upon the context. In the latter case, which we observed in the microchaete field of the pupal notum, the effect in the *neur*-overexpressing tissue was a zone of increased SOP density, with fairly regular spacing. Together, these data demonstrate the danger posed by persistent non-SOP expression of *neur*, resulting either in failure to establish the normal SOP fate or inappropriate specification of ectopic SOPs.

### Non-SOP activity of Neur is antagonized by BFM function

The above data establish both the existence of *neur* expression in multiple PNC cells prior to SOP specification and the danger posed by persistence of this expression in non-SOPs. Once SOP specification and effective inhibitory Notch signaling are established, the non-SOPs of the PNC prevent the accumulation of new *neur* transcript by deploying the E(spl)-C bHLH-Rs. But what about the Neur protein that is already present in non-SOPs due to the earlier *neur* expression? We hypothesized that the activity of this “ectopic” Neur protein is inhibited in non-SOPs by the Notch-dependent expression of the Bearded family proteins (BFMs), which bind directly to Neur and competitively block its interaction with the intracellular domains of Notch ligands, thus preventing any reciprocal signaling back to the SOP [15]. Consistent with this model, co-expression of *neur* and the BFM *E(spl)m4* using *mα-GAL4* significantly decreases the lateral inhibition disruptions caused by *neur* expression alone (Fig 5A). Conversely, we also assayed the effect of removing endogenous expression of two BFMs [*E(spl)mα* and *E(spl)m4*] on the *neur* misexpression phenotype. Adult flies homozygous for a double deletion of both *E(spl)mα* and *E(spl)m4* display a mild extra-bristle phenotype (Fig 5A). When *neur* is now misexpressed in this background using just a single copy of driver and responder, the number of extra bristles is greatly increased, far beyond that seen in a wild-type BFM background (Fig 5A). Thus, endogenous BFM expression in non-SOPs does strongly inhibit Neur activity in these cells. Of course, the severity of the extra-bristle phenotype in this experiment is artificially enhanced due to the high levels of Neur produced in response to the GAL4 driver. Therefore, we examined the consequence of loss of the two BFMs in a background homozygous for the *neurRC-4D*,*1BRm* rescue construct, which causes only a modest de-repression of *neur* in non-SOPs (Fig 3). Because the phenotypic effects vary substantially among different bristle positions, overall macrochaete counts on the head and thorax (Table 1) can be less informative than more focused assays. If we consider those bristle positions where we routinely observe ectopic reporter transgene activity or Neur::GFP expression, we see a statistically significant increase in bristle numbers in *E(spl)mα E(spl)m4* homozygous deletion flies with the addition of the Rm mutant *neur* rescue construct (Table 2).

**Table 1:** neurRC bristle counts.

Genotype (n = 50, unless noted)	Missing Bristles	Extra Bristles
*w*^*1118*^	0.08 ±0.05	0.22 ±0.07
*neurWT*.*V5*.*VK37*^a^	0.18 ±0.07	0.70 ±0.12
*neur4D*,*1B-RM*.*GFP*.*VK37*	0.40 ±0.11	0.28 ±0.08
*neurWT-attP40(#1)*^a^	0.00 ±0.00	0.62 ±0.12
*neur1B-RM-attP40(#1)*	0.04 ±0.04	0.64 ±0.12
*neur1B-RM-attP40(#2)*	0.04 ±0.03	0.88 ±0.14
*neur4D-RM-attP40*	0.00 ±0.00	0.30 ±0.08
*neur4D*,*1B-RM-attP40(#1)*	0.10 ±0.04	0.92 ±0.15
*neur4D*,*1B-RM-attP40(#2)*	0.12 ±0.05	0.60 ±0.11
*m4m*α	0.06 ±0.03	4.84 ±0.27
*neurWT*.*V5*.*VK37; m4m*α	0.04 ±0.03	3.24 ±0.30
*neur4D*,*1B-RM*.*GFP*.*VK37; m4m*α	0.12 ±0.06	2.36 ±0.23
*neurWT-attP40(#1); m4m*α *(n = 9)*	0.11 ±0.11	3.22 ±0.70
*neur1B-RM-attP40(#1); m4m*α *(n = 28)*	0.14 ±0.07	6.82 ±0.45
*neur4D-RM-attP40; m4m*α	0.46 ±0.09	1.54 ±0.21
*neur4D*,*1B-RM-attP40(#1); m4m*α	0.04 ±0.04	2.50 ±0.26
*neurWT-attP40 (line 1)/+; neur*^*IF65*^*/Df(3R)ED5330*	0.02 ±0.02	0.04 ±0.03
*neur1B-RM-attP40(#1)/+; neur*^*IF65*^*/Df(3R)ED5330*	0.02 ±0.02	0.06 ±0.03
*neur1B-RM-attP40(#2)/+; neur*^*IF65*^*/Df(3R)ED5330*	0.12 ±0.06	0.18 ±0.06
*neur4D-RM-attP40/+; neur*^*IF65*^*/Df(3R)ED5330*	0.03 ±0.03	0.18 ±0.07
*neur4D*,*1B-RM-attP40(#2)/+; neur*^*IF65*^*/Df(3R)ED5330 (n = 33)*	0.06 ±0.04	0.16 ±0.05

Bristle counts from the indicated genotypes, counting all macrochaete positions on the dorsal head and thorax, represented as mean ± SEM. Unless otherwise indicated, *neur* rescue construct insertions and/or mutant genotypes (e.g., *m4m*α) are homozygous.

^a^VK37 and attP40 denote ϕC31 docking sites.

**Table 2:** Dorsocentral and scutellar bristle counts.

Genotype	Missing Bristles	Extra Bristles
*w*^*1118*^	0.02 ±0.02	0.02 ±0.02
*neur4D*,*1B-Rm-attP40(#1)*	0.00 ±0.00	0.16 ±0.05
*m4m*α	0.00 ±0.00	0.44 ±0.09^a^
*neur4D*,*1B-Rm-attP40(#1); m4m*α	0.00 ±0.00	1.08 ±0.17^a^

Bristle counts from the indicated genotypes, counting only the dorsocentral and scutellar bristle positions. Statistical significance determined by pairwise ANOVA.

^a^*p*<0.01 comparing *m4m*α and *neur4D*,*1B-Rm; m4m*α.

### Neur function in the SOP inhibits *neur* expression in non-SOPs

**Fig 6:**
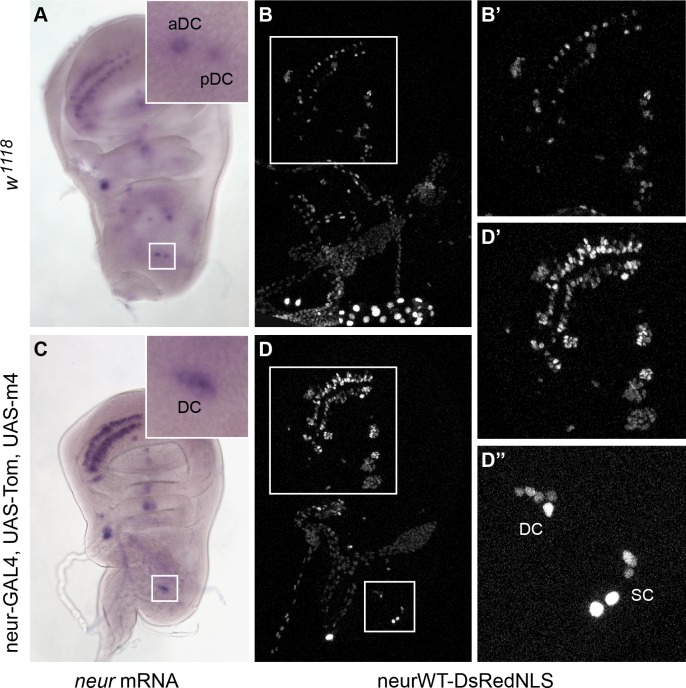
Inhibition of Neur function in the SOP causes ectopic *neur* transcript accumulation and neur4D enhancer activity. Comparison of the expression of *neur* mRNA (A and C) and of a neur4D-WT-DsRed reporter transgene (B and D) in wing imaginal discs from *w*^*1118*^ (A-B’) and *neur-GAL4*, *UAS-Tom*, *UAS-m4* flies (C-D”). Insets in A and C show higher-magnification views of the dorsocentral (DC) macrochaete cluster (boxed regions). B’ and D’ show higher-magnification views within the anterior wing pouch (regions boxed in B and D). D” shows a higher-magnification view of the dorsocentral and scutellar (SC) clusters (region boxed in D); compare with Fig 2.

To this point we have established that persistent *neur* expression in non-SOPs poses a threat to lateral inhibition, and have illuminated the mechanisms these cells use to antagonize the transcriptional activation (via the bHLH-Rs) and the function (via the BFMs) of *neur*. Since both of these non-SOP-specific inhibitors are direct targets of Notch signaling from the SOP, in which Neur is a critical cell-autonomous participant, it follows that blocking Neur function specifically in the SOP should lead to ectopic *neur* transcript accumulation in the other cells of the PNC. We therefore inhibited Neur function in the SOP by ectopically co-expressing two BFMs, *Tom* and *E(spl)m4*, in this cell using a *neur-GAL4* driver. As predicted, we observed in this genotype multiple positions in the wing imaginal disc displaying both ectopic *neur* transcript (Fig 6A and 6C) and ectopic expression of a neur4D reporter transgene (Fig 6B and 6B’ and 6D–6D”).

## Discussion

### Regulatory logic underlying the activation of *neur* transcription in SOPs

The logic of *neur* activation in SOPs appears remarkably complex. The presence of conserved proneural protein and bHLH-R binding motifs in neur4D and neur1B suggested that a simple “P+R” cis-regulatory code might underlie the operation of these enhancers—direct transcriptional activation by proneural proteins in the PNC, with non-SOP expression directly repressed by bHLH-Rs [13]. Mutating these motifs in the context of reporter transgenes, however, has revealed a more intricate regulatory scheme.

We observed that upon bHLH-R binding site mutagenesis in the *neur* enhancers, only a subset of non-SOP cells displayed ectopic expression. This contrasts with the behavior of previously studied SOP enhancers in the *phyllopod* (*phyl*) and *nervy* (*nvy*) genes, which exhibit strong and extensive de-repression in PNCs upon mutation of their bHLH-R motifs [13]. A number of circumstances may contribute to the weak de-repression of the *neur* enhancers. First, they may be subject to direct repression by additional factors beyond the bHLH-Rs. A strong precedent for this possibility is provided by the downstream SOP enhancer of the *senseless* (*sens*) gene, which is repressed in non-SOPs by both bHLH-Rs and the Sens protein itself [13]. Only when both of these inputs are eliminated does the enhancer exhibit substantial ectopic activity. Second, unlike the *phyl* SOP enhancer, the *neur* enhancers may be relatively unresponsive to the lower levels of proneural protein activity in non-SOP cells. Our finding that mutation of the proneural binding motifs in either *neur* enhancer fails to completely eliminate its SOP activity indicates that they both receive additional positive inputs, and these may be present at only marginal levels in non-SOPs. Since removal of Ac/Sc proneural activity in *trans* abolishes the activity of both enhancers [13], these additional factors most likely lie downstream of the proneurals in a coherent feed-forward regulatory structure [42].

Our results indicate that neur1B and neur4D are differentially dependent on the proneural component of this feed-forward mechanism. Mutating its proneural motifs has a stronger effect on neur1B’s activity, while neur4D likely relies more upon the proneural-dependent activation of several additional regulators. We suggest that SOP-specific enhancers that are targets of the proneurals typically lie at various positions along this spectrum, with their different requirements for direct proneural regulation possibly related to the timing of their activity or to the specific function of the associated gene during SOP specification and differentiation.

Other contrasts between neur1B and neur4D are also evident. There are marked differences in overall motif composition and organization; for example, neur4D contains two SMCα motifs, previously associated with activation in SOPs [29], while neur1B lacks them. In addition, the SOP-specific activity generated from the neur1B region of the locus seems to be distributed over a larger area, since a partially overlapping region, neur1C, also exhibits some weak SOP activity, and a larger fragment (NRS1) containing both neur1B and neur1C drives stronger and slightly expanded expression, including the wing margin [13]. By contrast, we have not detected enhancer activity in the intronic area adjacent to neur4D. Finally, it is noteworthy that neur1B and neur4D display a very different reliance on P_S_ versus P_A_ proneural binding motifs.

Overall, the many structural and functional differences between neur1B and neur4D may reflect a role for the two enhancers in ensuring the robustness of *neur*’s expression in SOPs [43, 44]. While these modules exhibit a largely overlapping SOP functionality [13], it may be advantageous for them to rely differentially on various positive and negative inputs in order to better withstand a range of genetic and environmental perturbations.

### Utilization of common proneural protein binding motifs by Ac/Sc and Ato

The evolutionary appearance of distinct Atonal and Achaete/Scute subfamilies of proneural proteins likely predates the cnidarian/bilaterian divergence, perhaps 550–600 Mya [33, 45]. It is perhaps not surprising, therefore, that Ato and Ac/Sc factors have been found to have distinct roles in cell fate specification during development. In *Drosophila*, for example, the external sensory organs of the peripheral nervous system are dependent on *ac/sc* gene function, while chordotonal organs and the R8 photoreceptors of the eye rely on *ato* [46]. Despite this, it is certainly reasonable to imagine—given their shared role in the overall process of neurogenesis—that the target gene repertoires of the Ato and Ac/Sc factors might be substantially overlapping, and indeed many common targets have been identified. In some instances, the two factor types have been found to regulate a common target largely via distinct binding sites, as exemplified by the *Brd* gene [47, 48]. By contrast, we have shown here that *neur* utilizes proneural binding motifs of the CAGATG class to mediate activation by both Ac/Sc and Ato. The logic underlying the use of common versus distinct proneural sites in the same target is not entirely clear, but may reflect constraints imposed by selective interactions with regulatory cofactors [46].

### Activation of *neur* transcription in a “pre-SOP” subset of the PNC

Previous studies of *neur* expression and function in PNCs during lateral inhibition have relied on reporter genes [13, 37, 38] or mutational analysis [13, 20, 23]. Our direct analysis of *neur* transcription and protein accumulation in macrochaete PNCs has demonstrated explicitly that, prior to SOP specification, a distinctive subset of PNC cells activates *neur* expression.

Lack of *neur* function during Notch-mediated lateral inhibition results in a comparatively modest mutant phenotype by comparison to the effects of losing the activity of other “neurogenic” genes such as *Notch* itself [20]. Specifically, only a relatively small subset of cells in the PNC commit inappropriately to the SOP fate [20, 23]. We suggest that these ectopic SOPs correspond to the “pre-SOP” subset identified here by *neur* expression analysis, and thus that the “pre-SOPs” overlap strongly, or even coincide, with the “*neur* group” described by Troost et al. [23].

Given the essential role—both direct and indirect—played by proneural gene activity in activating *neur* expression [13, 24], it is likely that this is the principal determinant of which PNC cells are members of the “pre-SOP” group. Thus, the “pre-SOPs” would correspond to those cells with the highest levels of net proneural activity—the cells with the highest levels of proneural protein accumulation and the lowest levels of expression of the inhibitory Extramacrochaetae (Emc) protein [49].

### Two levels of cell-non-autonomous, negative autoregulation of *neur* function

The need to specify only one SOP cell within each PNC presents clear regulatory challenges. The very fact that membership in the PNC is defined by expression of proneural factors imposes the strict requirement that the net levels of proneural activity in the non-SOP cells be kept below a threshold that would permit their inappropriate commitment to the SOP fate. Likewise, it is critical that the non-SOPs—either individually or collectively—do not become sufficiently strong Notch signalers as to inhibit the proper specification of the single SOP. Since Neur is a principal determinant of this signaling capacity, it is vital that only the SOP acquires sufficient Neur activity to become a fully effective signal source. Yet *neur* transcription is both directly and indirectly activated by proneural factors, and while this gives the SOP a clear advantage (due to its elevated level of proneural protein), it also creates the serious risk of one or more non-SOPs developing inappropriately high levels of Neur function.

**Fig 7:**
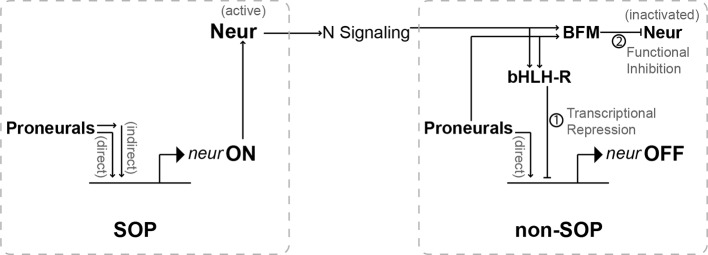
Through N signaling, proneural-dependent SOP expression of Neur promotes the inhibition of both *neur* transcription and Neur function in non-SOP cells. Proneural proteins activate *neur* transcription both directly, via binding sites in the neur4D and neur1B enhancers, and indirectly by activating expression of other positive regulators of *neur* in the SOP. *neur*-dependent N signaling, combined with proneural factor activity, non-autonomously promotes expression of both *E(spl)* bHLH-Rs and BFMs in non-SOP cells. The bHLH-Rs repress further transcription of *neur* directly, through binding motifs in neur4D and neur1B, and similarly inhibit the expression of other SOP-specific targets. The BFMs bind Neur and block its interaction with Dl, preventing non-SOP cells from sending an effective N signal back to the SOP.

We have shown here that the lateral inhibition network utilizes two distinct mechanisms to counter this threat. The first operates at the level of controlling *neur* transcription in non-SOPs (Fig 7). Notch signaling from the SOP activates the expression of multiple Hes-class bHLH repressor proteins specifically in the non-SOPs [35]. These factors are thus ideally suited to the task of inhibiting the expression of SOP genes only in non-SOPs [13]. Direct transcriptional repression of *neur* by the Hes proteins works, then, to counteract the proneural-dependent activation of the gene in non-SOPs.

However, the threat of inappropriate Neur activity in non-SOPs has a second source (Fig 7). We have demonstrated that, prior to the establishment of effective Notch signaling activity by the presumptive SOP (and therefore prior to the onset of Hes repressor function in non-SOPs), a subset of non-SOP cells (the “pre-SOPs”) actively transcribe *neur*. The resulting *neur* mRNAs could then encode sufficient Neur protein to confer significant Notch signaling capacity on one or more pre-SOPs, potentially resulting in inhibition of the SOP’s fate commitment. This possibility is countered by a second class of Notch pathway targets, the *Brd* gene family, transcription of which is likewise activated selectively in non-SOPs [35]. As potent direct inhibitors of Neur’s function in activating Notch ligands [14, 15, 50], the Brd proteins offer an effective post-transcriptional solution to the problem of Neur protein accumulation in non-SOPs.

Due to the essential role it plays in establishing the SOP’s Notch signaling capacity, Neur is indirectly responsible for stimulating the expression in non-SOPs of both the Hes repressors and the Brd proteins, both of which act to antagonize Neur activity in these cells (Fig 7). It follows, then, that the *neur* gene engages in two distinct modes of cell-non-autonomous negative autoregulation during lateral inhibition, which serve to insure the robustness of the SOP specification process.

## Materials and methods

### Fly strains and mosaic analysis

The *E(spl)mα-Gal4* driver was described previously [35]. *UAS-neur* and *UAS-Tom* were constructed by Eric Lai, and *UAS-FLAGm4* by Joseph Fontana [15]. The *E(spl)mα E(spl)m4* double-deletion line was a generous gift from Joseph Fontana, constructed via two independent homologous recombination events using the methods described [51]. Stocks for generating *neur* MARCM clones (*y w hs-FLP*^*122*^ *tub-Gal4 UAS-GFP-6xnls; FRT82B tub-Gal80/TM6B* and *w; FRT82B neur*^*1*^ *cu/TM6B*) were generously provided by Christos Delidakis [20]. *UAS-neur* was crossed in to create the stock *w; UAS-neur; FRT82B neur*^*1*^ *cu/TM6B*. Mosaic analyses using the FLP/FRT and MARCM systems have been described [20, 52–55].

### Reporter constructs

Reporter constructs for neur4DWT (primers 5'-CCAAGACCCAAATTTAGTTGGTATTCAAGC-3' and 5'-AATAGGCCCCAATCCAGTACACGTATGTGC-3') and mutants (P_S_ and P_A_, RCANNTG>RAANNGG; Sens, AAATCTGT>AGGTCTGT; bHLH-R, CACGYG>CCCTYT; SMC, AGGGGTTG>AAAAAAAA; for “mystery blocks,” all nucleotides in S2 Fig converted to A) were cloned into pH-Stinger [56] or pH-RedStinger [57]. Mutations were generated by overlap extension PCR [58]. At least three independent transformant lines were analyzed before a representative line was selected for all further analysis. Constructs were injected using standard transformation techniques [59], with *w*^*1118*^ as the recipient strain. Wild-type (primers NRS1B-u 5'-TCCCAGTTTTGAAACCATTAGCTTACACAG-3' and NRS1B-d 5'-AAAGACAATTGTGAGGCCAGAGGGTAATGC-3') and mutant versions of neur1B were generated and cloned into pH-Stinger-attB and injected using the ΦC31 integrase system [60] into the docking site VK00037 [36]. The neur4D and neur1B variants in S5 Fig, as well as the constructs from the promoter-proximal regions shown in S1 Fig, were cloned into pH-Stinger-attB and integrated into the ΦC31 docking site attP2 (1B-C: NRS1B-u and NRS1C-d; 1C: NRS1C-u 5'-GCAGACAGCTGCTTCCATTTGCATTTGTCG-3' and NRS1C-d 5'-ATTCCCTTTTGTGTCCGCAGGATTAGTTCG-3'; 1BC: NRS1BC1.1-u 5'-TCGATATCCACTGTACCCATCATGATCACC-3' and NRS1BC1.1-d 5'-GCAAAGGTAGTAACTCGATCGTAATGGAGG-3'; 1BBC: NRS1B-u and NRS1BC1.1-d).

### Rescue constructs

neurRC-WT-P[acman] constructs were generated by BACR09F04-mediated gap repair of attB-P[acman]-AmpR via recombineering, as described [36]. The region cloned extends to the Eag I sites on either side of the *neur* locus (from sequence CGGCCGCCTCCAGGATAAGATGCT to sequence GATATACCCGCTGTGAATCGGCCG, a 21-kb region). These constructs were subsequently injected into the docking sites attP40 and attP16 [61] by Genetic Services, Inc., using the ΦC31 integrase system [60]. Mutant and tagged variants of this starting construct were generated by recombineering using galK-mediated selection [62], and injected into the attP40 docking site. neurRC-WT-GFP was integrated into the attP40, attP2, attP16, and VK00037 docking sites [36, 61, 63]; neurRC-4D,1BRm-GFP was integrated into VK00037 for comparison with the WT-GFP at the same site.

### *In situ* hybridization

Single-probe *in situ* hybridizations were performed as previously described [10, 24, 64, 65]. Quantification of *in situ* signal area and darkness for the neurRC-4D,1BRm experiment was performed using ImageJ software, taking the average of 9 discs for the WT construct and 21 discs for the Rm construct. Statistical significance was assayed by ANOVA. Multiplex fluorescent *in situ* hybridizations in third-instar wing imaginal discs were performed basically as described [39]; anti-hapten antibodies (sheep anti-DIG, mouse anti-biotin, and chicken anti-DNP) were used at a 1:5000 dilution in 1X PBS + 0.1% Triton X-100 (PBT), without using a block solution (we observed too much background in disc tissue when using the Roche Block mentioned in Kosman *et al*.). Probes were constructed by cloning an intronic DNA fragment into pGEM-T, linearizing, and transcribing RNA using the T7 RNA polymerase following the Kosman protocol. The following probes were used: DNP-*sca*, DIG-*neur*, BIO-*neur*, BIO-*CG32150*. Images were captured as described below, adjusting the gain to maximally reveal any coincidence between *neur* probes.

### Immunohistochemistry

With the exception of GFP antibody staining, immunohistochemistry was performed essentially as described previously [64]. Discs from neurRC-WT-GFP discs also included a blocking step after fixation in 0.3% milk in PBT. Blocking was done overnight at 4°C, with primary antibodies added the next morning, also in the milk blocking solution. Secondary antibodies for this stain were added in PBT only. The following antibodies were used: guinea pig anti-Sens (generously provided by Hugo Bellen), 1:2000; mouse anti-Cut (2B10) [Developmental Studies Hybridoma Bank (DSHB), University of Iowa], 1:100; rabbit anti-GFP (Invitrogen), 1:500. All secondary antibodies used were AlexaFluor varieties from Invitrogen and included anti-rabbit-Alexa488 conjugate, anti-guinea pig-Alexa555 conjugate, anti-mouse-Alexa555 conjugate, and anti-mouse-Alexa647 conjugate. Secondaries were always used in staining at a 1:1000 dilution in PBT. For the fluorescent *in situ* hybridizations, the secondaries were all raised in donkey.

### Analysis of GFP reporter expression and bristle phenotypes

Multiple independent transformant lines were collected for each pH-Stinger GFP reporter construct. Imaginal discs from at least 10 larvae were collected for each line and analyzed for variation across the line. To record images, imaginal discs from at least 10 larvae or pupae carrying wild-type and mutant constructs were collected, dissected and fixed, and imaged in parallel under identical confocal settings. Representative images are displayed in the figure panels. For analysis of ectopic GFP reporter expression due to transcription factor binding motif mutations (Fig 2), 30 third-instar larvae were dissected and all discs with discernible DC and SC positions were analyzed, noting the presence of any cell expressing nuclear GFP but not Sens at these positions.

For quantification of bristle phenotypes (Fig 5; both Tables), all macrochaete positions on the dorsal head and thorax were analyzed, and each position scored for either missing or extra bristles, over a total of 25 males and 25 females unless otherwise noted. Statistical significance was determined by pairwise ANOVA.

### Confocal microscopy

Confocal microscopy procedures have been described previously [64]. Images of fluorescent *in situ* hybridizations were collected as series of 1-micron sections; antibody stains were collected at low magnification as 2-micron sections, with high-magnification images as 1-micron sections. For the collection of z-sections to generate the cross-sectional view shown in Fig 4E, we shortened the distance to 0.75-micron sections. Images were collected using Leica confocal software, cropped with Adobe Photoshop, and combined into figures using Adobe Illustrator.

### Gene structure and sequence alignment diagrams

Gene structure and sequence alignment diagrams were constructed using the latest version of the GenePalette software tool (http://www.genepalette.org) [66] and were edited in Adobe Illustrator.

### Primers

Additional oligonucleotide primer sequences are available upon request.

## Supporting information

S1 Text Supporting materials and methods. Electrophoretic mobility shift assays (EMSAs).
(PDF)

S1 Fig Two *neuralized* SOP enhancers contain conserved binding sites for both proneural and E(spl)-C bHLH-R transcription factors. Diagram of the *neur* locus and flanking genes shows the locations of the neur4D and neur1B enhancer regions. Above and below the diagram are graphical alignments representing neur4D (A) and neur1B (B). Identical sequences > 8 bp are connected by solid vertical lines. Sequence identities inverted relative to *D*. *melanogaster* are represented as red lines. A phylogenetic tree is included for reference at the left of the species names in A. Also shown in A is the span of the neurA construct [67]. See also S2 Fig. In B, note that the entire neur1B enhancer region has undergone an inversion event since the last common ancestor of the obscura and melanogaster groups. Also in B, the sequence TTTTGTCAGC was used to track P4 through its change from P_S_ to P_A_, as well as its inversion.
(TIF)

S2 Fig Sequence alignments of conserved motifs in the neur4D and neur1B enhancers. (B) Diagram of the *neur* locus and flanking genes shows the locations of the neur4D and neur1B enhancer regions. Immediately above and below the gene diagram are lines representing the neur4D (above) and neur1B (below) regions from *D*. *melanogaster*, denoting the locations of the conserved motifs. Regions with > 8 bp of sequence identity are marked on the lines with gray boxes. (A, C) Alignments of sequence motifs within (A) neur4D and (C) neur1B, labeled as in B. Non-conserved flanking nucleotides are also shown, in lighter text. Sequences inverted relative to *D*. *melanogaster* are displayed in red. In the case of proneural motifs where the majority of species match the RCAGSTG (P_S_) definition, the mismatched nucleotide is underlined in the divergent species. Species in which a sequence orthologous to the P1 (P_S_) or the P_A_ site in neur4D has not been identified are omitted from that alignment.
(TIF)

S3 Fig Localizing SOP enhancer activity in the promoter-proximal region of *neur*. (A) Diagram of the *neur* locus, showing the locations and boundaries of the regions assayed for enhancer activity in this study. (B-F) Representative third-instar wing imaginal discs illustrating the capacity of the promoter-proximal reporter constructs to drive an SOP expression pattern. (B) NRS1B-C>GFP, (C) NRS1B>GFP, (D) NRS1C>GFP, (E) NRS1BBC>GFP and (F) NRS1BC>GFP.
(TIF)

S4 Fig Effects of motif mutagenesis in the neur4D enhancer. (A) Mutation of single motif classes in wing imaginal discs (1–14), 12 hr APF nota (15–21), and 24 hr APF nota (22–28). (B) Mutation of the same motif classes represented in A, along with mutation of P_S_ proneural protein binding motifs. GFP signal is in green; Sens protein signal is in magenta. Asterisk in A8 denotes the observation of a GFP-positive, Sens-negative cell adjacent to a GFP-negative, Sens-positive cell. Carets in A11 point to ectopic GFP-positive, Sens-negative cells. Panels 8–14 in both A and B show higher-magnification views of the dorsocentral and scutellar macrochaete clusters (boxed in panels 1–7).
(TIF)

S5 Fig Analysis of the effects of neur4D motif mutations in embryos. Shown are representative *in situ* hybridizations in embryos using either a probe for *neur* (top row) or a probe for GFP (remaining rows).
(TIF)

S6 Fig Characterization of CAGATG sequences as functional binding sites for proneural proteins. (A) Electrophoretic mobility shift assay showing that GST-Sc/GST-Da and GST-Ato/GST-Da heterodimers bind efficiently to specific E-box sequences from the neur1B enhancer region, but not to the mutated versions of these sequences. BrdE3 probe [48] is used as a positive control for Atonal binding [47]. We note that we have consistently observed little or no binding of GST-Sc/GST-Da to BrdE3 (see also Singson *et al*. [48]), in contrast to other reports [47]. Box on the right displays sequence segments containing the putative proneural binding motifs, their difference(s) from the P_S_ motif definition (highlighted in red), and the nucleotide changes in the mutant probes. (B-G) Third-instar larval tissues displaying expression differences between neur1BWT>GFP (B-D) and neur1BP_S+A_m>GFP (C-G) reporter constructs. (H-R) Third-instar imaginal discs bearing different neur4D>GFP reporter variants, comparing neur4DWT>GFP (H, M, and N), neur4DP_S_m>GFP (I, O, and P), neur4DP_S+A_m>GFP (J, Q, and R), neur4D(P_S_+SMC+Sens+MB2)m>GFP (K), and neur4D(P_S+A_+SMC+Sens+MB2)m>GFP (L).
(TIF)
