# Supplementary material for: Lateral inhibition: Two modes of non-autonomous negative autoregulation by neuralized
Source: PLoS Genet. 2018 Jul 20;14(7):e1007528. doi: 10.1371/journal.pgen.1007528 (PMC6070291; doi:10.1371/journal.pgen.1007528)
Supplement: S1 Text — Electrophoretic mobility shift assays (EMSAs). (PDF) [file pgen.1007528.s001.pdf]

Supporting materials and methods

Electrophoretic mobility shift assays (EMSAs)

500-ml cultures of BL21 cells expressing GST-Ato, GST-Sc, and GST-Da [1] were grown at 37°C until an Abs600 of 0.6 was reached. Expression was induced at 1 mM IPTG for 2 hours at 30°C. Cell pellets were resuspended in ice-cold 1X PBS with protease inhibitor tablet (Roche) and lysed with 1 mg/ml lysozyme for 30 min on ice. After sonication (3X, 1 min each) DTT was added to 5 mM, RNAse A to 10 μg/ml, DNAse I to 250 μg/ml, and Triton X-100 to 1%. Lysate was rocked at 4°C for 30 min, then pelleted at 12,000g for 10 min at 4°C. Cleared lysate was incubated with pre-washed glutathione agarose beads (GE Healthcare) for 30 min at 4°C. Beads were washed 3X with ice-cold 1X PBS and once with ice-cold Elution Wash (50 mM Tris pH 9.5, 1% Triton X-100, 5 mM DTT). Protein was eluted 1 ml at a time with Elution Wash + 10 mM glutathione, pH 8.5. Glycerol was added to each elution to 20% and protein concentration was estimated by comparison to BSA standards via SDS-PAGE.

30-mer oligonucleotides were labeled with Biotin-11-UTP and annealed in vitro according to manufacturer's instructions (Thermo Scientific). Quantities of GST-Sc, GST-Ato, and GST-Da were optimized based on binding to labeled control probes, to approximately 60 ng for each protein. Protein and oligonucleotides (20 pmol labeled) were incubated at room temperature for 20 min, then run on 5% non-denaturing polyacrylamide gels (BioRad). Free and bound labeled oligonucleotides were detected using the LightShift Chemiluminescent EMSA kit, according to manufacturer's instructions (Thermo Scientific).

1. Singson A, Leviten MW, Bang AG, Hua XH, Posakony JW. Direct downstream targets of proneural activators in the imaginal disc include genes involved in lateral inhibitory signaling. Genes Dev. 1994; 8: 2058-2071.
