# Supplementary figures and images for: Lateral inhibition: Two modes of non-autonomous negative autoregulation by neuralized
Source: PLoS Genet. 2018 Jul 20;14(7):e1007528. doi: 10.1371/journal.pgen.1007528 (PMC6070291; doi:10.1371/journal.pgen.1007528)

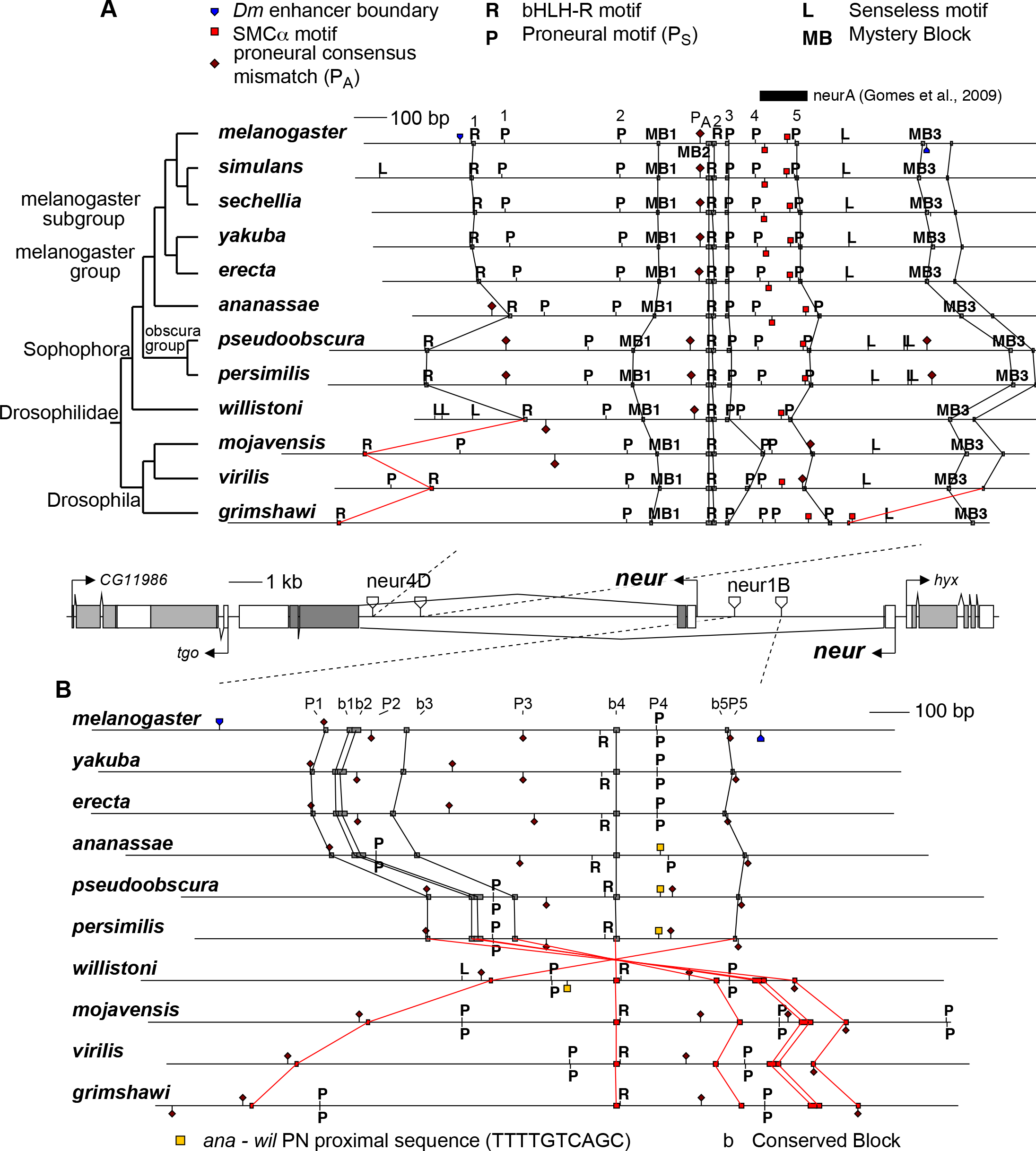

Supplement: S1 Fig — Diagram of the neur locus and flanking genes shows the locations of the neur4D and neur1B enhancer regions. Above and below the diagram are graphical alignments representing neur4D (A) and neur1B (B). Identical sequences > 8 bp are connected by solid vertical lines. Sequence identities inverted relative to D. melanogaster are represented as red lines. A phylogenetic tree is included for reference at the left of the species names in A. Also shown in A is the span of the neurA construct [67]. See also S2 Fig. In B, note that the entire neur1B enhancer region has undergone an inversion event since the last common ancestor of the obscura and melanogaster groups. Also in B, the sequence TTTTGTCAGC was used to track P4 through its change from PS to PA, as well as its inversion. (TIF) [file pgen.1007528.s002.tif]

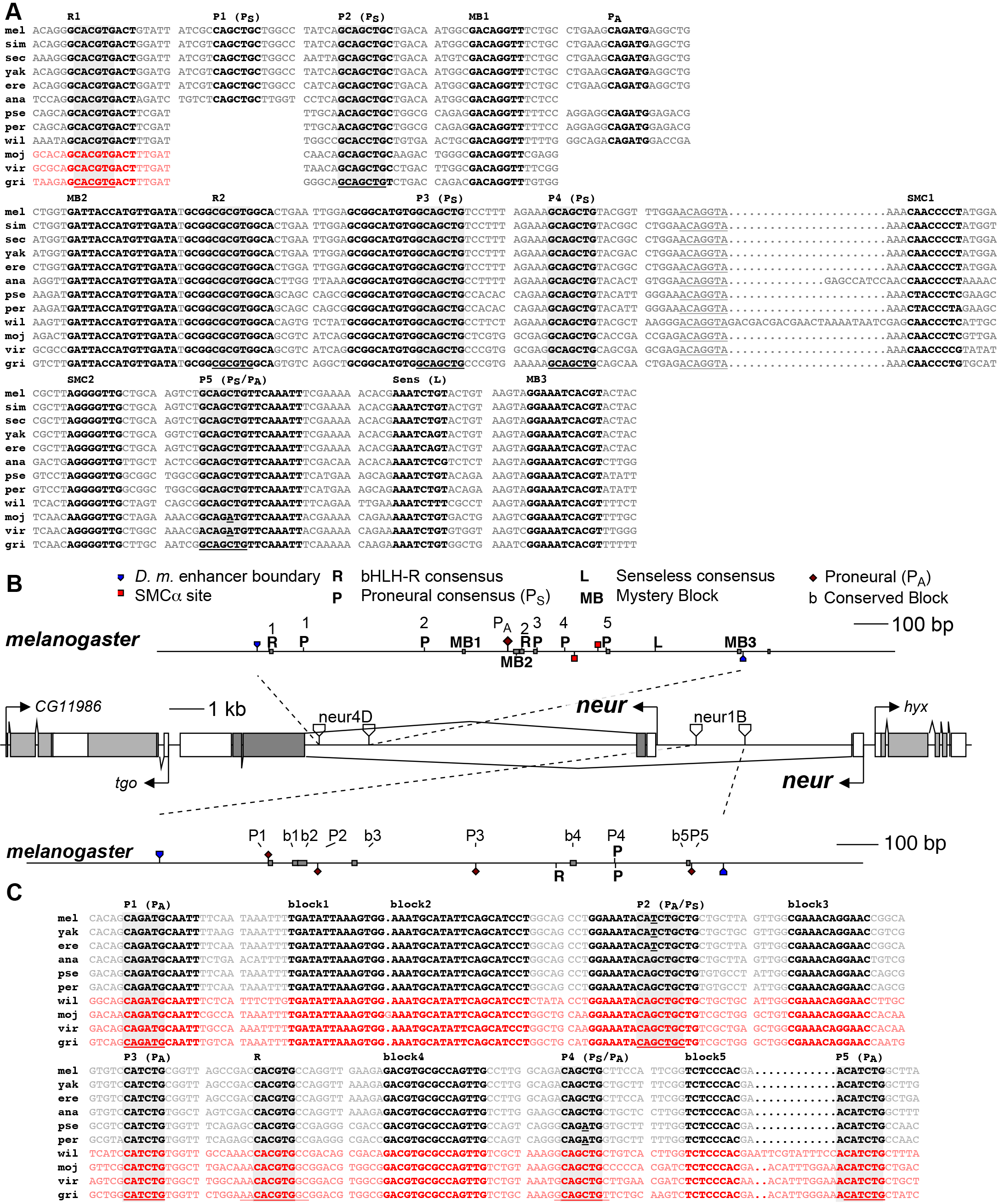

Supplement: S2 Fig — (B) Diagram of the neur locus and flanking genes shows the locations of the neur4D and neur1B enhancer regions. Immediately above and below the gene diagram are lines representing the neur4D (above) and neur1B (below) regions from D. melanogaster, denoting the locations of the conserved motifs. Regions with > 8 bp of sequence identity are marked on the lines with gray boxes. (A, C) Alignments of sequence motifs within (A) neur4D and (C) neur1B, labeled as in B. Non-conserved flanking nucleotides are also shown, in lighter text. Sequences inverted relative to D. melanogaster are displayed in red. In the case of proneural motifs where the majority of species match the RCAGSTG (PS) definition, the mismatched nucleotide is underlined in the divergent species. Species in which a sequence orthologous to the P1 (PS) or the PA site in neur4D has not been identified are omitted from that alignment. (TIF) [file pgen.1007528.s003.tif]

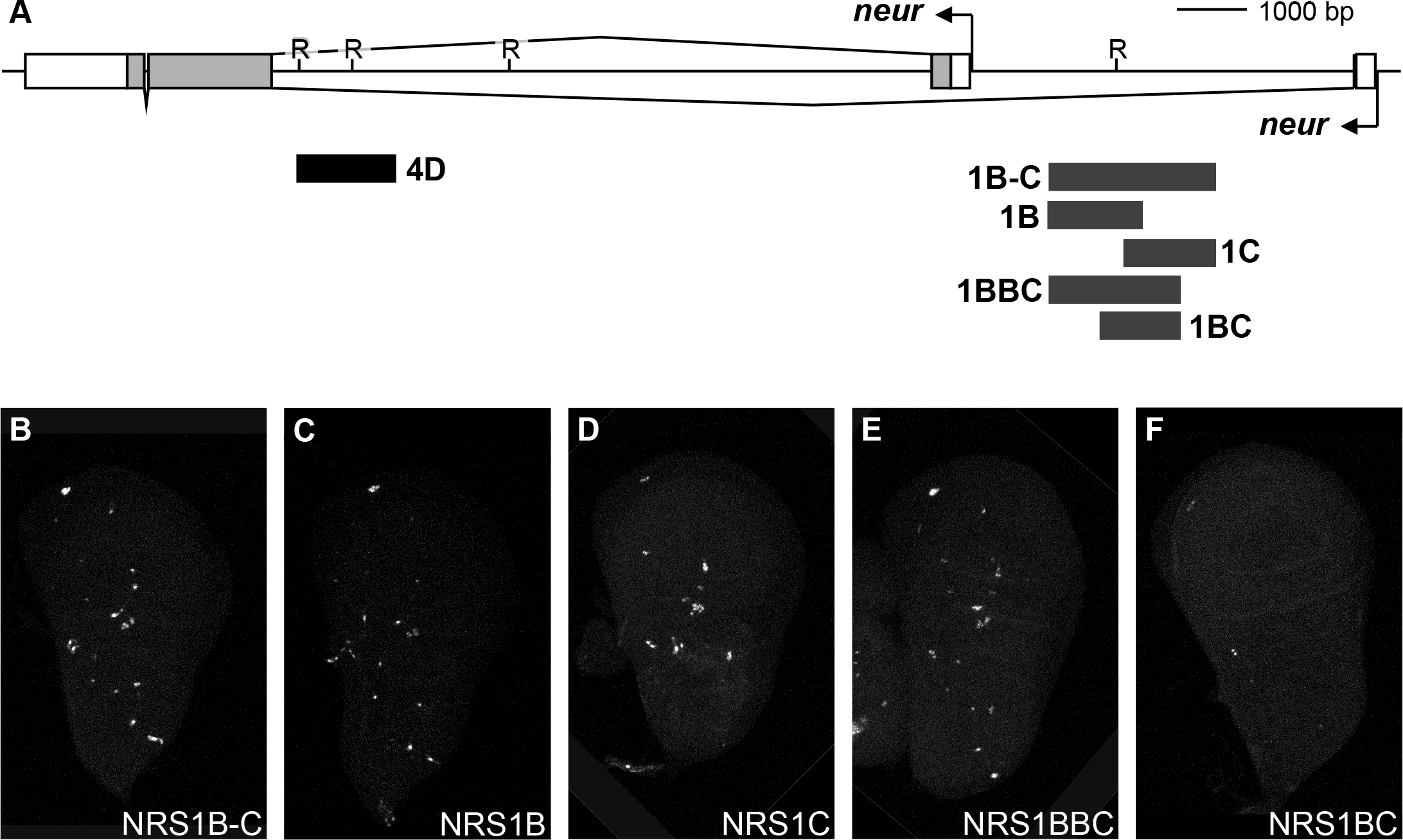

Supplement: S3 Fig — (A) Diagram of the neur locus, showing the locations and boundaries of the regions assayed for enhancer activity in this study. (B-F) Representative third-instar wing imaginal discs illustrating the capacity of the promoter-proximal reporter constructs to drive an SOP expression pattern. (B) NRS1B-C>GFP, (C) NRS1B>GFP, (D) NRS1C>GFP, (E) NRS1BBC>GFP and (F) NRS1BC>GFP. (TIF) [file pgen.1007528.s004.tif]

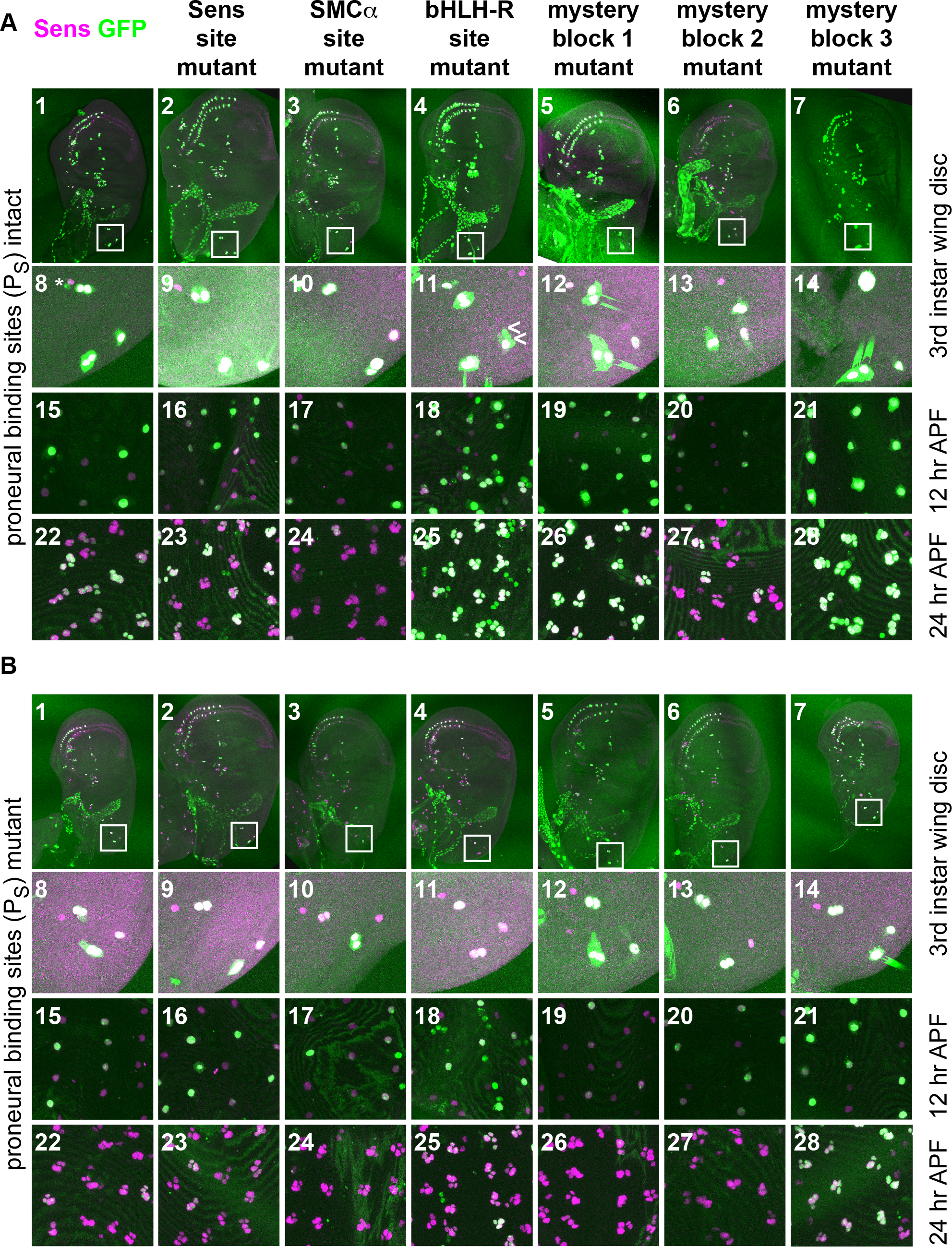

Supplement: S4 Fig — (A) Mutation of single motif classes in wing imaginal discs (1–14), 12 hr APF nota (15–21), and 24 hr APF nota (22–28). (B) Mutation of the same motif classes represented in A, along with mutation of PS proneural protein binding motifs. GFP signal is in green; Sens protein signal is in magenta. Asterisk in A8 denotes the observation of a GFP-positive, Sens-negative cell adjacent to a GFP-negative, Sens-positive cell. Carets in A11 point to ectopic GFP-positive, Sens-negative cells. Panels 8–14 in both A and B show higher-magnification views of the dorsocentral and scutellar macrochaete clusters (boxed in panels 1–7). (TIF) [file pgen.1007528.s005.tif]

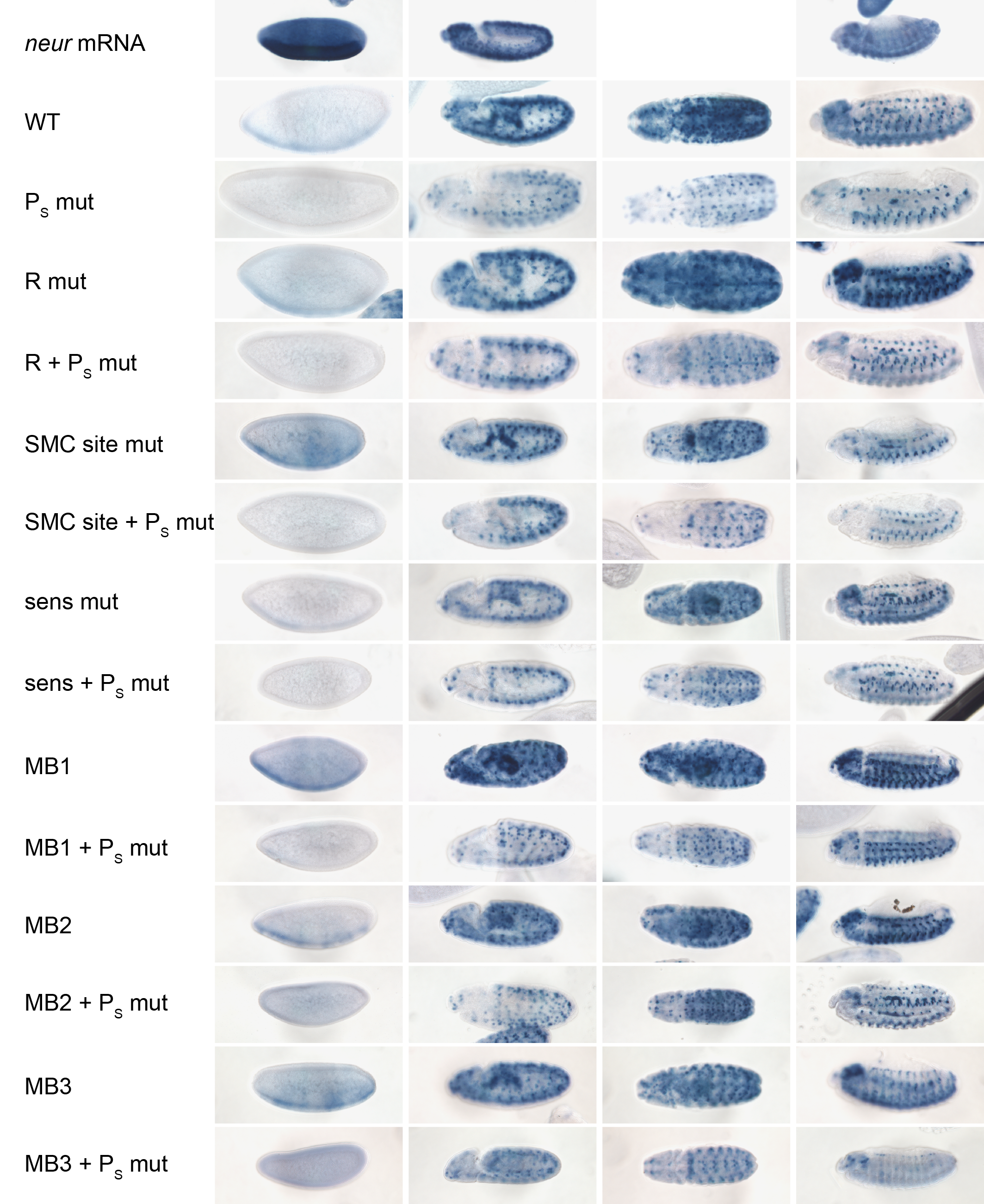

Supplement: S5 Fig — Shown are representative in situ hybridizations in embryos using either a probe for neur (top row) or a probe for GFP (remaining rows). (TIF) [file pgen.1007528.s006.tif]

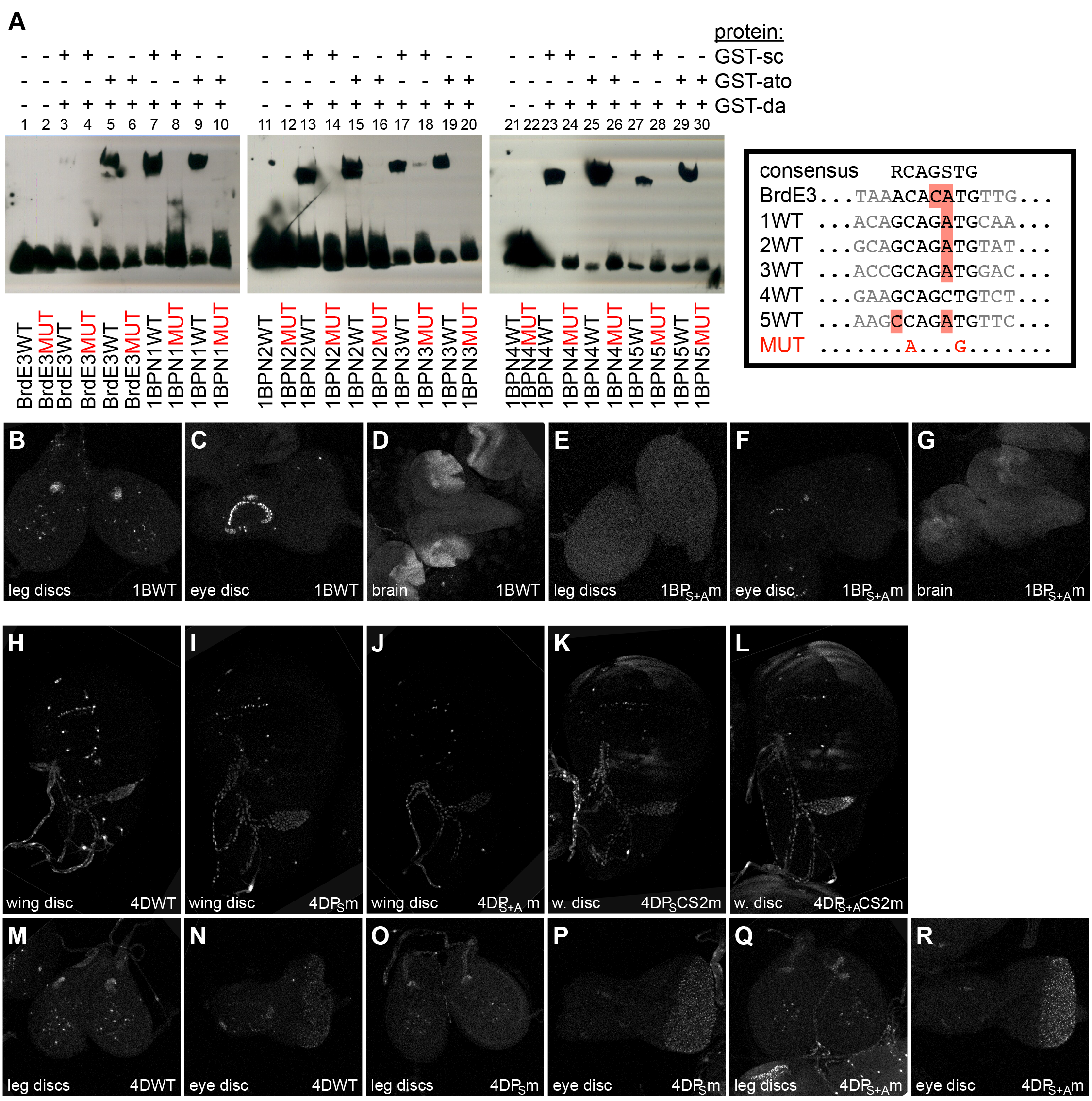

Supplement: S6 Fig — (A) Electrophoretic mobility shift assay showing that GST-Sc/GST-Da and GST-Ato/GST-Da heterodimers bind efficiently to specific E-box sequences from the neur1B enhancer region, but not to the mutated versions of these sequences. BrdE3 probe [48] is used as a positive control for Atonal binding [47]. We note that we have consistently observed little or no binding of GST-Sc/GST-Da to BrdE3 (see also Singson et al. [48]), in contrast to other reports [47]. Box on the right displays sequence segments containing the putative proneural binding motifs, their difference(s) from the PS motif definition (highlighted in red), and the nucleotide changes in the mutant probes. (B-G) Third-instar larval tissues displaying expression differences between neur1BWT>GFP (B-D) and neur1BPS+Am>GFP (C-G) reporter constructs. (H-R) Third-instar imaginal discs bearing different neur4D>GFP reporter variants, comparing neur4DWT>GFP (H, M, and N), neur4DPSm>GFP (I, O, and P), neur4DPS+Am>GFP (J, Q, and R), neur4D(PS+SMC+Sens+MB2)m>GFP (K), and neur4D(PS+A+SMC+Sens+MB2)m>GFP (L). (TIF) [file pgen.1007528.s007.tif]
